# Treatment of Obstinate Cystitis

**Published:** 1902-03

**Authors:** George W. Hopkins

**Affiliations:** Cleveland, Ohio


### Treatment of Obstinate Cystitis.

By GEORGE W. HOPKINS, M. D., Cleveland, Ohio.

John C., age 31, occupation, patrolman. Following exposure, patient experienced bladder symptoms as follows: frequent urination, tenesmus, hypogastric pain and temperature of 101.4°. The urine was scanty, turbid and loaded with mucus.

*Diagnosis.*—Acute cystitis. Treatment consisted of rest in bed, restricted diet, anodynes for the tenesmus, diluent and alkaline drinks. The acute symptoms promptly subsided, but the urine continued abnormal despite the general measures employed and the internal administration of urinary antiseptics. Irrigation with boric acid solutions of various strength proved unsatisfactory, as did also solutions of potassium permanganate and silver nitrate, similarly applied. A 20 per cent. solution of glyco-thymoline was substituted for irrigation, and the improvement was marked and continuous until recovery was perfect.

Harry R., age 43; occupation, bookkeeper. Had a history of bladder trouble of several years duration. His urine was blood-tinged and loaded with mucus. Microscopic examination revealed an abundance of ammonia, magnesium phosphates, numerous disintegrating pus corpuscles, blood corpuscles and blood shadows.

Repeated examination with the sound gave negative results, but a skiograph taken with a high vacuum hard tube revealed a small calculus which had persistently evaded the sound in previous examinations. Lithotomy was performed and the calculus removed but the urine failed to return to normal. Irrigation in turn with boric acid, potassium permanganate and silver nitrate solutions gave unsatisfactory results. Glyco-thymoline irrigations proved satisfactory from the start and recovery was ultimately perfect.

William L., age 55; occupation, saloonkeeper. Had a history of repeated attacks of gonorrhea which were never appropriately treated. Urine was voided with great difficulty, at frequent intervals and loaded with mucus. Reaction was alkaline and the microscope revealed an abundance of amorphous phosphates of calcium and magnesium, flat epithelial cells, disintegrating pus corpuscles and indigo crystals. Examination confirmed diagnosis of chronic cystitis due to urethral stricture and hypertrophied prostate.

Catelectrolysis by the slow method removed the stricture and Bottini's operation relieved the enlarged prostate, but the urine failed to clear up as desired. The cystoscope showed marked changes in the bladder walls, but catheterisation of the ureters yielded negative results. Appropriate urinary antiseptics were administered internally and silver nitrate solutions by vesical irrigation with only slight improvement. Irrigation with 20 per cent. solutions of glyco-thymoline gave early and continuous improvement until recovery was perfect.

